# Interleukin-3 enhances the migration of human mesenchymal stem cells by regulating expression of CXCR4

**DOI:** 10.1186/s13287-017-0618-y

**Published:** 2017-07-14

**Authors:** Amruta Barhanpurkar-Naik, Suhas T. Mhaske, Satish T. Pote, Kanupriya Singh, Mohan R. Wani

**Affiliations:** National Centre for Cell Science, S. P. Pune University Campus, Pune, 411 007 India

**Keywords:** Interleukin-3, Mesenchymal stem cells, Cell migration, CXCR4, SDF-1α

## Abstract

**Background:**

Mesenchymal stem cells (MSCs) represent an important source for cell therapy in regenerative medicine. MSCs have shown promising results for repair of damaged tissues in various degenerative diseases in animal models and also in human clinical trials. However, little is known about the factors that could enhance the migration and tissue-specific engraftment of exogenously infused MSCs for successful regenerative cell therapy. Previously, we have reported that interleukin-3 (IL-3) prevents bone and cartilage damage in animal models of rheumatoid arthritis and osteoarthritis. Also, IL-3 promotes the differentiation of human MSCs into functional osteoblasts and increases their in-vivo bone regenerative potential in immunocompromised mice. However, the role of IL-3 in migration of MSCs is not yet known. In the present study, we investigated the role of IL-3 in migration of human MSCs under both in-vitro and in-vivo conditions.

**Methods:**

MSCs isolated from human bone marrow, adipose and gingival tissues were used for in-vitro cell migration, motility and wound healing assays in the presence or absence of IL-3. The effect of IL-3 preconditioning on expression of chemokine receptors and integrins was examined by flow cytometry and real-time PCR. The in-vivo migration of IL-3-preconditioned MSCs was investigated using a subcutaneous matrigel-releasing stromal cell-derived factor-1 alpha (SDF-1α) model in immunocompromised mice.

**Results:**

We observed that human MSCs isolated from all three sources express IL-3 receptor-α (IL-3Rα) both at gene and protein levels. IL-3 significantly enhances in-vitro migration, motility and wound healing abilities of MSCs. Moreover, IL-3 preconditioning upregulates expression of chemokine (C-X-C motif) receptor 4 (CXCR4) on MSCs, which leads to increased migration of cells towards SDF-1α. Furthermore, CXCR4 antagonist AMD3100 decreases the migration of IL-3-treated MSCs towards SDF-1α. Importantly, IL-3 also induces in-vivo migration of MSCs towards subcutaneously implanted matrigel-releasing-SDF-1α in immunocompromised mice.

**Conclusions:**

The present study demonstrates for the first time that IL-3 has an important role in enhancing the migration of human MSCs through regulation of the CXCR4/SDF-1α axis. These findings suggest a potential role of IL-3 in improving the efficacy of MSCs in regenerative cell therapy.

**Electronic supplementary material:**

The online version of this article (doi:10.1186/s13287-017-0618-y) contains supplementary material, which is available to authorized users.

## Background

Human mesenchymal stem cells (MSCs) have been exploited for their regenerative potential to repair damaged tissues in various animal models and also in some human diseases [1, 2]. Ease of isolation of MSCs from adult tissues without much ethical concern and their possible ex-vivo expansion, multidifferentiation potential and, most importantly, immunosuppressive and immunomodulatory properties make them a suitable candidate for cell therapy in regenerative medicine [3, 4]. In addition, MSCs possess migratory capacity and ability to secrete different cytokines and growth factors that help in tissue regeneration [3]. These studies suggest that MSCs hold great promise for the success of regenerative cell therapies. However, most systemically infused MSCs are trapped in the lungs, raising controversy regarding their beneficial effects due to lack of site-directed migration and poor engraftment [5–7]. It is also reported that in the absence of ex-vivo pretreatment or modification, fewer infused MSCs migrate and actually engraft at damaged tissue [8, 9]. The biodistribution of MSCs into multiple organs in nonhuman primates, experimental laboratory animals and human beings is also reported [10–12]. However, there is limited evidence for efficient migration and homing of infused MSCs towards injured or damaged tissues [13]. Thus, clinical success of MSC therapy depends on their efficient migration towards damaged tissues.

The mechanism(s) involved in migration and homing of exogenously infused MSCs towards damaged tissues is not fully understood. The possible involvement of stromal cell-derived factor-1 alpha (SDF-1α)/chemokine (C-X-C motif) receptor 4 (CXCR4) interaction in MSC migration has been reported [13–15]. Also, activation of the CXCR4/SDF-1α axis is believed to be a crucial pathway in migration of MSCs towards bone marrow (BM), brain, kidney and other injured tissues [13, 16–18]. However, CXCR4 is expressed only on a small population of MSCs and its expression decreases further during ex-vivo expansion, which may reduce the cells’ ability to respond to homing signals emerging from damaged tissues [13, 19]. Thus, targeting CXCR4 could be an important strategy to improve the in-vivo migration efficiency of MSCs.

Various strategies to improve migration, homing and engraftment of MSCs to damaged tissues have been reported. Genetic modification with the incorporation of homing molecules such as CXCR4 or the α4 subunit of very late antigen-4 integrin is shown to enhance migration and homing potential of MSCs [20–22]. The enzymatic modification of native CD44 on the MSC membrane into hematopoietic cell E-selectin/L-selectin ligand showed their increased migration towards BM [23]. Although genetically and enzymatically modified MSCs have shown encouraging results in cell therapies, they address serious safety concerns. Migratory capacity of MSCs is also regulated by various soluble factors including inflammatory cytokines such as TNF-α and IL-1β. Pretreatment of MSCs with TNF-α induces their migration towards injured tissue [24]. Similarly, IL-1β pretreatment enhances the migration of MSCs in an experimentally induced murine model of colitis [25]. However, TNF-α and other inflammatory cytokines promote apoptosis of infused MSCs and inhibit bone regeneration [26]. The short-term exposure of fetal Flk^+^ BM-MSCs with a cocktail of cytokines upregulates CXCR4 expression and induces their migration in irradiated NOD/SCID mice [27]. Nevertheless, this cocktail contains a high concentration of cytokines that may be harmful. Thus, factors that could promote the efficient migration, homing and regenerative potential of MSCs are lacking.

Interleukin-3 (IL-3), a cytokine secreted by activated T lymphocytes, is known to regulate hematopoiesis. Previously, we have reported that IL-3 prevents bone and cartilage damage in animal models of human rheumatoid arthritis and osteoarthritis [28, 29]. IL-3 also promotes the differentiation of human MSCs into functional osteoblasts and increases their in-vivo regenerative potential in immunocompromised mice [30]. However, the role of IL-3 in migration and motility of MSCs is not yet known. In this study, we investigated the role of IL-3 on migration of human MSCs isolated from BM, adipose tissue (AT) and gingival tissue (GT). We found that IL-3 significantly enhances the migration, motility and wound healing abilities of MSCs by upregulating the expression of chemokine receptor CXCR4. Moreover, IL-3-induced CXCR4 expression leads to increased migration of MSCs towards SDF-1α. Interestingly, IL-3 induces in-vivo migration of human MSCs towards matrigel-releasing SDF-1α in immunocompromised mice. Thus, we revealed that in addition to its role in regenerative potential, IL-3 has a novel role in enhancing the migration and homing efficiency of MSCs. Our results indicate the therapeutic potential of IL-3 in enhancing the efficacy of MSCs in regenerative medicine.

## Methods

### Animals

NOD/SCID mice 6–12 weeks old were obtained from the Experimental Animal Facility of National Centre for Cell Science, Pune, India. All experiments involving animal use were approved by the Institutional Animal Ethics Committee of National Center for Cell Science, Pune, India.

### Isolation and expansion of human MSCs

All study protocols for using human samples were approved by the Institutional Ethics Committee of the National Center for Cell Science. MSCs from human BM (BM-MSCs) were isolated as described previously [30]. Briefly, BM aspirates were washed to remove adipose tissues. Nucleated cells were obtained by density gradient centrifugation using Ficoll-Hypaque (Sigma-Aldrich, St. Louis, MO, USA), washed and resuspended in DMEM-low glucose supplemented with 10% fetal calf serum (FCS; Life Technologies, Rockville, MD, USA).

MSCs from human GT (GT-MSCs) were isolated as described previously [31]. Briefly, GT was washed in PBS and the epithelial cell layer was removed. Tissue was minced into small pieces and incubated in medium containing 0.1% collagenase and 0.2% dispase (MP BioMedicals, Santa Ana, CA, USA) for 15 minutes at 37 °C. The first cell fraction of enzyme digestion containing epithelial cells was discarded. Tissue was further incubated with enzyme solution for 15 minutes. The cells were washed and resuspended in DMEM-low glucose with 10% FCS.

Human AT samples were obtained from individuals undergoing abdominal liposuction surgery. MSCs from AT (AT-MSCs) were isolated using the method described previously [32]. Briefly, lipo-aspirates were washed three or four times with medium and incubated with 0.2% collagenase for 120 minutes at 37 °C with gentle agitation in a shaking water bath. Collagenase was inactivated with 10% FCS, followed by washing to remove remaining oil and fat globules from the stromal vascular fraction. The pelleted stromal vascular fraction was used as a source of MSCs and resuspended in alpha-MEM containing 10% FCS.

MSCs isolated from all three sources were seeded at a density of 2 × 10^5^–4 × 10^5^ cells/cm^2^ and after 72 hours nonadherent and loosely adherent cells were discarded. Adherent cells were washed thoroughly and cultures were fed every 2–3 days with respective medium. Cells were subcultured at 80% confluence using trypsin phosphate versene glucose. Cells from passages 2–4 were used in all experiments.

### Flow cytometry

BM-MSCs, AT-MSCs and GT-MSCs were characterized for the presence of surface markers by flow cytometry [33]. Briefly, cells were washed with FACS buffer (PBS containing 0.5% bovine serum albumin), fixed with 4% paraformaldehyde (pH 7.4) for 5 minutes and blocked with 1% human serum in PBS for 30 minutes. Cells were incubated with fluorochrome-labeled antibodies for CD90, CD44, CD29, CD45, CD34, CD73, CD105, MHC class I and class II (BD Biosciences, Mountain View, CA, USA) and respective isotypes (1:100) for 45 minutes. Cells were washed three times with FACS buffer, acquired on FACS-Canto (BD Biosciences) and analyzed using FACS Diva software (BD Biosciences). A similar procedure was used for surface staining of MSCs with antibodies for different chemokine receptors (CCR1, CCR7, CCR9, CX3CR1, CXCR5, CXCR6) and integrins (α4, α5, all from Biolegend, San Diego, CA, USA), and CXCR4, CXCR7 and IL-3 receptor α (R&D Systems, Minneapolis, MN, USA). Intracellular staining of cells was performed after permeabilization using 0.1% Triton-X-100.

### Confocal microscopy

Human MSCs (10^3^ cells/well) were cultured on coverslips in 24-well plates. After 24 hours cells were washed in PBS, fixed in 4% paraformaldehyde, blocked with 1% human serum for 30 minutes and incubated with purified anti-human-IL-3Rα antibody (Santa Cruz Biotechnology Inc., CA, USA), followed by FITC-conjugated secondary antibody (1:100; Abcam, Cambridge, UK). The coverslips were mounted on glass slides and observed under a Zeiss LSM 510 META confocal microscope (Zeiss, Jena, Germany).

### RT-PCR and real-time PCR

RNA was isolated using TRIzol reagent (Life Technologies) and cDNA was prepared (cDNA synthesis kit; Life Technologies). RT-PCR was performed using Thermal Cycler (Eppendorf, Hamberg, Germany). Each cycle of RT-PCR consisted of 35 cycles of 30 seconds of denaturation at 94 °C, 30 seconds of annealing at 60 °C and 30 seconds of extension at 72 °C. RT-PCR primers used were IL-3Rα F-5′-GGAGAATCTGACCTGCTGGA-3′ and R-5′-ACTTTGAGAACCGCTGGAGA-3′, and β-actin F-5′-CGGGAAATCGTGCGTGACAT-3′ and R-5′-ATCTTCATTGTGCTGGGTGCC-3′. Quantitative real-time PCR was performed using Universal PCR mix and Taqman primers and probe (Applied Biosystems, CA, USA) for human CXCR4 (Hs00607978_s1) and β-actin (Hs00664172_s1) on a StepOnePlus machine (Applied Biosystems). The reaction consisted of 30 seconds of annealing at 60 °C for 40 cycles. Fold change in expression of genes was calculated relative to β-actin levels by the comparative ∆Ct method.

### Cell migration, motility and wound healing assays

Migration and wound healing assays were performed using cell culture inserts (pore size 8.0 μm, 24-well format; BD Falcon) and monolayer cultures respectively [34]. Briefly, IL-3-pretreated (100 ng/ml for 24 hours) or untreated human MSCs (2 × 10^5^ cells/insert) in 200 μl of serum free-media were added to the upper chamber of cell culture inserts and SDF-1α (Peprotech, Rehovot, Israel) in 500 μl of serum-free media was added to the lower chamber. In the case of the blocking experiment, IL-3-treated cells were incubated in the upper chamber with CXCR4 antagonist AMD3100 (10 μM; Sigma-Aldrich). After 18 hours, cells from the upper side of inserts were carefully removed with a cotton swab. The lower side of the inserts was then stained with hematoxylin and observed under the phase-contrast microscope (Zeiss). For quantitative analysis, migrated cells on the lower side of inserts were removed by trypsinization and counted. Data are expressed as the number of cells migrating towards SDF-1α.

For the wound healing assay, 10^4^ cells/well were plated in 24-well plates and allowed to form a monolayer. The wounds were created by scratching monolayers with a 200 μl pipette tip. The cell layer was washed thoroughly and incubated further for 18 hours with or without IL-3 and/or AMD3100. Images were captured at 0 and 18 hours using the phase-contrast microscope. Wound healing was quantified by calculating the area of the wound at 0 and 18 hours, using the “MRI Wound healing tool” in Image J software (open source, NIH, USA). Percent wound closure was calculated as described previously [35] using the following formula:

$$ \%\ \mathrm{Wound}\ \mathrm{closure} = \frac{\mathrm{Wound}\ \mathrm{area}\ \left({\mathrm{T}}_0\right)\ \hbox{--}\ \mathrm{Wound}\ \mathrm{area}\ \left({\mathrm{T}}_{18}\right)}{\mathrm{Wound}\ \mathrm{area}\ \left({\mathrm{T}}_0\right)}\kern0.5em  \times 100 $$

For assessment of cell motility, MSCs (2 × 10^3^ cells/well) were cultured for 24 hours in six-well plates. Time-lapse microscopic imaging of cells was initiated immediately after addition of IL-3 (100 ng/ml) and was carried over 24 hours in a controlled biochamber at 5% CO_2_ and 37 °C. Images were acquired every 10 minutes by a Nikon ECLIPSE TE2000-E Microscope (Japan). Thirty randomly chosen cells were analyzed in three independent experiments. Cell tracking was performed using Image J software (open source, NIH, USA) including the plug-ins “Manual Tracking” (Fabrice Cordelières, France) and “Chemotaxis and Migration Tool” (IBIDI Integrated BioDiagnostics, Germany). Cell coordinates were retrieved by marking the nuclei in every captured frame. The data were further processed, visualized and plotted, thereby allowing the calculation of migration as accumulated and euclidean distances [36].

### In-vivo cell migration assay

In-vivo assessment of MSC migration was performed using a modified matrigel plug assay as described previously [32]. NOD/SCID mice were divided into five groups: control-uninjected, untreated BM-MSCs, IL-3-treated BM-MSCs, untreated AT-MSCs and IL-3-treated AT-MSCs (*n* = 6 per group). Matrigel (BD Pharmingen) was mixed with SDF-1α (100 ng/ml). Each mouse received 300 μl matrigel containing SDF-1α per implant (two implants subcutaneously on the right dorsal side) and matrigel without SDF-1α (two implants subcutaneously on the left dorsal side). After 2 hours, IL-3-treated or untreated MSCs (10^5^ cells in 50 μl PBS) were prelabeled using Qtracker™ 655 (Life Technologies) and injected subcutaneously at the center, equidistant from all four implants. Mice were acquired on the In Vivo Imaging System (IVIS Spectrum; PerkinElmer, Waltham, MA, USA) at 0 hours for detection of labeled MSCs and at 24 hours for detection of migrated MSCs towards the matrigel plugs. All mice were then sacrificed and matrigel plugs were harvested. Cells were isolated from matrigel plug by melting matrigel at 4 °C and acquired on FACS Canto for detection of labeled cells.

### MTT, in-vivo toxicity and tumorogenicity assays

See Additional file 1: Supplementary Methods for details of MTT, in-vivo toxicity and tumorogenicity assays.

### Statistical analysis of data

Data are expressed as mean ± SEM. An unpaired two-tailed Student *t* test was applied for statistical analysis between the groups. Nonparametric data were compared by Mann–Whitney test. The significance values are defined as *p* ≤ 0.05, *p* ≤ 0.01 and *p* ≤ 0.001.

## Results

### Expression of IL-3Rα on human MSCs

**Fig. 1:**
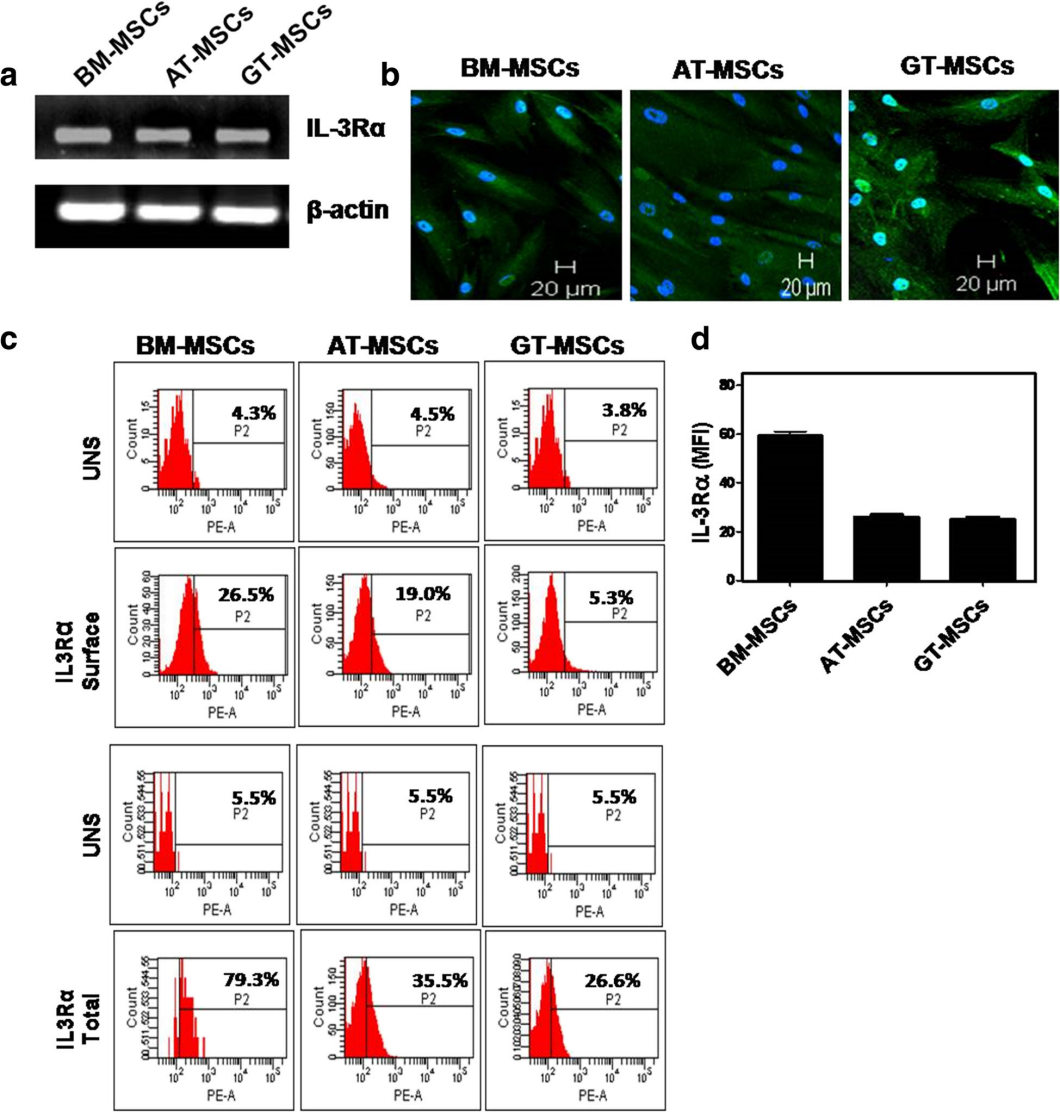
Human MSCs express IL-3Rα. Human BM-MSCs, AT-MSCs and GT-MSCs of passage 2 were subjected to RT-PCR (**a**), confocal (**b**, magnification 10×) and flow cytometry (**c**) analysis to examine the expression of IL-3Rα both at mRNA and protein levels. Graphical representation of mean fluorescent intensity (*MFI*) of IL-3Rα on human MSCs (**d**). Similar results were obtained in two independent experiments. *AT* adipose tissue, *BM* bone marrow, *GT* gingival tissue, *IL-3R*α interleukin-3 receptor alpha, *MSC* mesenchymal stem cell

We reported earlier that human BM-MSCs express IL-3Rα [30]. To evaluate the role of IL-3 on migration of human MSCs derived from various sources, expression of IL-3Rα was also confirmed on MSCs derived from other two sources such as human AT and GT along with BM. All three sources of human MSCs used in this study were a homogeneous population from passages 2–4. We observed that all of these MSCs express IL-3Rα at a transcriptional level (Fig. 1a). The expression of IL-3Rα at protein level was also confirmed by immunocytochemistry and flow cytometry. Figure 1b, c shows the surface as well as intracellular expression of IL-3Rα on MSCs derived from three different sources. Although the mRNA expression of IL-3Rα on three sources of MSCs was similar, a substantial difference in their protein expression was observed by flow cytometry. BM-MSCs and AT-MSCs showed higher expression of IL-3Rα than GT-MSCs both at surface and intracellular levels. Figure 1d shows the mean fluorescence intensity of IL-3Rα on MSCs. It was observed that IL-3Rα expression was higher in BM-MSCs than AT-MSCs and GT-MSCs. This variation in surface expression of IL-3Rα could be because of the different sources of MSCs, individual donor variation and culture conditions. Thus, we confirmed that human MSCs derived from various sources express IL-3Rα both at gene and protein levels.

### Effect of IL-3 on wound healing and cell motility of MSCs

**Fig. 2:**
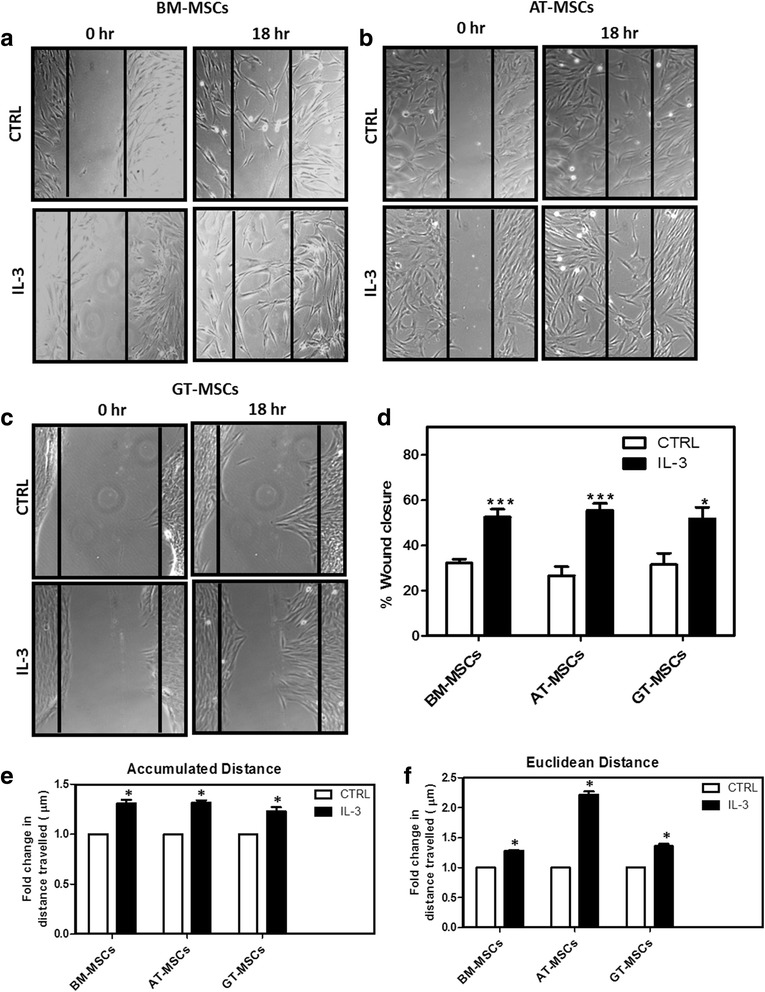
Effect of IL-3 on wound healing and cell motility of human MSCs. BM-MSCs, AT-MSCs and GT-MSCs (10^4^ cells/well) were seeded in 24-well culture plates. After 80–90% confluency, wounds were created on monolayers using a 200 μl pipette tip. Cells were washed and incubated for 18 hours in the absence or presence of human IL-3 (100 ng/ml). Representative images of human BM-MSCs (**a**), AT-MSCs (**b**) and GT-MSCs (**c**) at 0 and 18 hours of IL-3 treatment (Magnification 10×). Percent wound closure from three independent experiments was analyzed (**d**). Human BM-MSCs, AT-MSCs and GT-MSCs were incubated for 24 hours with and without IL-3 and cell motility was examined by measurement of accumulated (**e**) and euclidean (**f**) distance travelled by MSCs. The cell motility images were captured by time-lapse microscope and analyzed using Image J Software. Data shown as mean ± SEM of three independent experiments. *p ≤ 0.05 and ***p ≤ 0.001 vs control group. *AT* adipose tissue, *BM* bone marrow, *CTRL* control, *GT* gingival tissue, *IL-3* interleukin-3, *MSC* mesenchymal stem cell

The effect of IL-3 on migration ability of MSCs was evaluated using an in-vitro wound healing assay that mimics cell migration in vivo [34]. The wounds created on monolayers of BM-MSCs, AT-MSCs and GT-MSCs were treated with IL-3 (100 ng/ml) for 18 hours. It was observed that, as compared to control, a greater number of IL-3-treated MSCs migrated from the edge of the wound towards the wound area. The migratory effect of IL-3 was seen in MSCs derived from all three sources (Fig. 2a–c). Calculation of percent wound healing revealed that IL-3 significantly enhances wound closure in all three sources of MSCs (Fig. 2d).

To further evaluate the effect of IL-3 on cell motility, all three MSCs were subjected to time-lapse video microscopic analysis as described in Methods. Computation of accumulated and euclidean distances of MSCs from their positions at the 0 time point to the end time point illustrates the cell motility and displacement, respectively. Figure 2e, f shows that accumulated as well as euclidean distances traveled by MSCs were significantly increased by IL-3. The euclidean distance traveled by AT-MSCs in the presence of IL-3 was greater than that of BM and GT-MSCs. The results obtained by cell motility assay are consistent with those obtained by wound healing assay, suggesting that IL-3 has the potential to induce both migration and motility of human MSCs. 

### IL-3 increases CXCR4 expression on MSCs

**Fig. 3:**
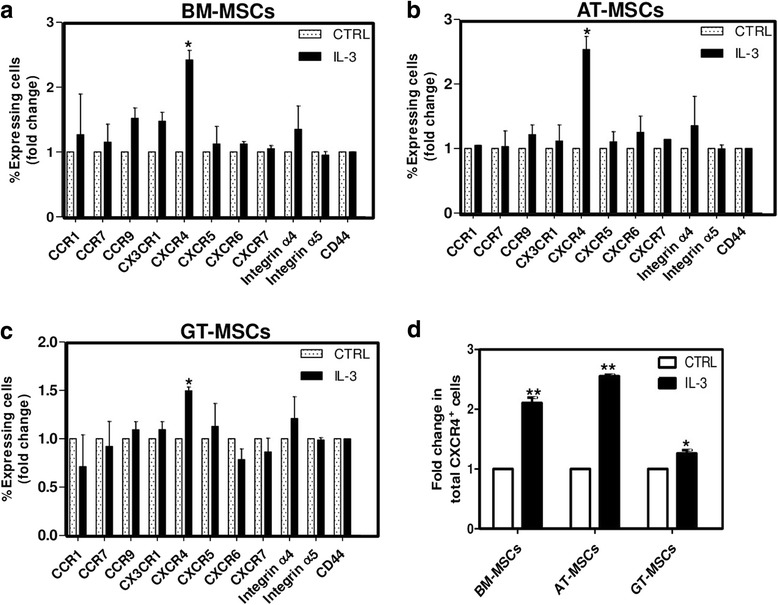
Effect of IL-3 on expression of chemokine receptors and integrins involved in migration of MSCs. BM-MSCs, AT-MSCs and GT-MSCs were treated with IL-3 (100 ng/ml) for 24 hours and surface expression of different chemokine receptors and integrins was analyzed by flow cytometry. **a**–**c** Fold change as percentage of cells expressing chemokine receptors and integrins. **d** Fold change in percentage of cells expressing intracellular CXCR4 analyzed after IL-3 treatment in permeabilized MSCs. Data shown as mean ± SEM of three independent experiments. *p ≤ 0.05 and **p ≤ 0.01 vs control groups. *AT* adipose tissue, *BM* bone marrow, *CTRL* control, *GT* gingival tissue, *IL-3* interleukin-3, *MSC* mesenchymal stem cell

To investigate the mechanism of enhanced wound healing and motility of MSCs in the presence of IL-3, we pretreated BM-MSCs, AT-MSCs and GT-MSCs with IL-3 (100 ng/ml) for 24 hours and the expression of integrins (α4 and α5), chemokine receptors (CCR1, CCR7, CCR9, CX3CR1, CXCR4, CXCR5, CXCR6, CXCR7) and CD44 molecules involved in cell migration was analyzed by flow cytometry. Although IL-3 treatment enhances the surface expression of many chemokine receptors, CXCR4 expression was significantly and consistently increased in all three MSCs. In BM-MSCs and AT-MSCs, IL-3 treatment increased CXCR4 expression by more than 2-fold; whereas a 1.5-fold increase was observed in GT-MSCs (Fig. 3a–c). In all MSCs, integrins α4 and α5 and CD44 expression did not show any significant change after IL-3 treatment. Because CXCR4 is an important and most documented chemokine receptor responsible for MSC migration, its surface as well as intracellular expression in presence of IL-3 was analyzed by flow cytometry. Total CXCR4 protein expression in IL-3-treated MSCs was significantly upregulated by more than 2-fold (Fig. 3d).

**Fig. 4:**
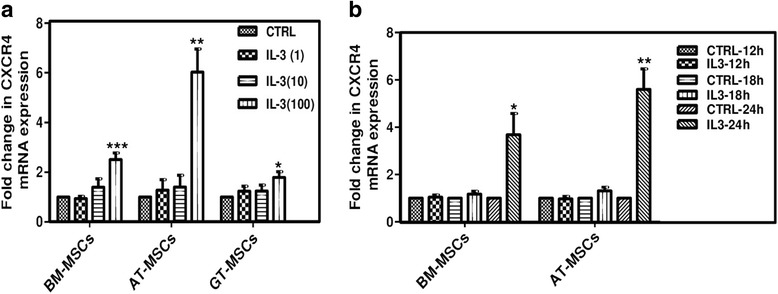
Effect of IL-3 on mRNA expression of CXCR4 in MSCs. BM-MSCs, AT-MSCs and GT-MSCs were treated with different concentrations of IL-3 for 24 hours and fold change in mRNA expression of CXCR4 was analyzed using real-time PCR (**a**). CXCR4 mRNA expression in MSCs incubated with IL-3 (100 ng/ml) was analyzed after 12, 18 and 24 hours (**b**). Data shown as mean ± SEM of three independent experiments. *p ≤ 0.05, **p ≤ 0.01 and ***p ≤ 0.001 vs control groups. *AT* adipose tissue, *BM* bone marrow, *CTRL* control, *CXCR4* chemokine (C-X-C motif) receptor 4, *GT* gingival tissue, *IL-3* interleukin-3, *MSC* mesenchymal stem cell

Increased CXCR4 expression on MSCs by IL-3 was also confirmed at mRNA level by incubating BM-MSCs, AT-MSCs and GT-MSCs for 24 hours with different concentrations of IL-3 and was analyzed by quantitative real-time PCR. It was observed that IL-3 (100 ng/ml) significantly increased CXCR4 expression in all MSCs. There was an approximately 3-fold increase in CXCR4 expression in BM-MSCs, a 6-fold increase in AT-MSCS and a 1.5-fold increase in GT-MSCs (Fig. 4a). All further experiments were conducted using human BM-MSCs and AT-MSCs. The effect of IL-3 on CXCR4 mRNA expression was also studied in a time-dependent manner by incubating BM-MSCs and AT-MSCs with IL-3 (100 ng/ml) for 12, 18 and 24 hours. We found that CXCR4 mRNA expression was significantly increased by IL-3 at 24 hours (Fig. 4b). Thus, IL-3 upregulates CXCR4 expression in MSCs both at gene and protein levels.

### Comparative effect of different cytokines on regulation of CXCR4

**Fig. 5:**
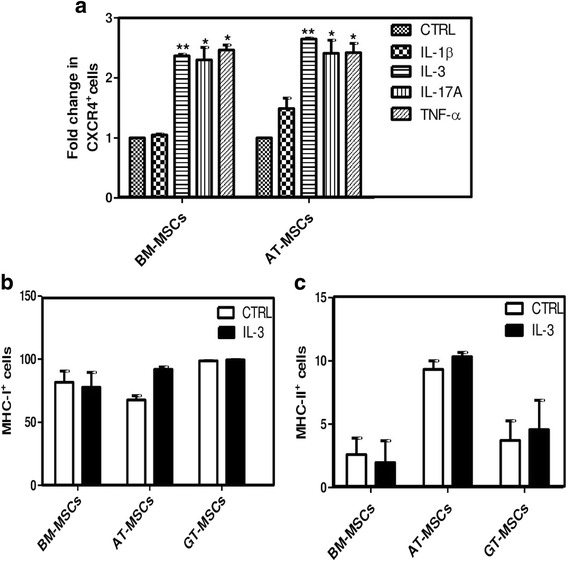
Comparison of effect of different cytokines on regulation of CXCR4. To compare the induction of CXCR4 expression by different cytokines, BM-MSCs and AT-MSCs were incubated with IL-1β (10 ng/ml), IL-3 (100 ng/ml), IL-17A (50 ng/ml) and TNF-α (50 ng/ml) independently for 24 hours. Fold change in percentage of CXCR4^+^ cells was analyzed by flow cytometry (**a**). Expression of MHC class I (**b**) and class II (**c**) molecules on MSCs was analyzed by flow cytometry after IL-3 treatment for 24 hours, and compared with respective untreated MSCs. Data shown as mean ± SEM of three independent experiments. *p ≤ 0.05 and **p ≤ 0.01 vs control groups. *AT* adipose tissue, *BM* bone marrow, *CTRL* control, *CXCR4* chemokine (C-X-C motif) receptor 4, *GT* gingival tissue, *IL-3* interleukin-3, *MSC* mesenchymal stem cell

Proinflammatory cytokines such as IL-1β and TNF-α have been reported to increase the migration of MSCs [24, 25]. Also, IL-17A enhances immunosuppressive and immunomodulatory properties of human MSCs [37]. Therefore, we compared the effect of IL-3 with other cytokines on CXCR4 expression in MSCs by flow cytometry. BM-MSCs and AT-MSCs were incubated with IL-3 (100 ng/ml), IL-17A (50 ng/ml), IL-1β (10 ng/ml) and TNF-α (50 ng/ml) independently for 24 hours. The surface expression of CXCR4 was increased up to 2-fold by IL-3, IL-17A and TNF-α, whereas IL-1β did not show any significant change in its expression (Fig. 5a). The CXCR4 induction by IL-3 was comparable to that of IL-17A and TNF-α.

Pretreatment of human MSCs with IFN-γ enhances their immunosuppressive properties but makes them immunogenic due to elevated expression of MHC class II molecule [38]. To investigate the effect of IL-3 on MSC phenotypic marker expression, cells were incubated with IL-3 (100 ng/ml) for 24 hours and subjected to flow cytometry analysis for expression of MHC class I and II molecules along with other phenotypic markers. We found that IL-3 pretreatment does not affect the expression of MHC class I and class II molecules on all MSCs (Fig. 5b, c) and maintained their normal phenotype (Additional file 1: Figure S1A, S1B). These results suggest that IL-3 has no effect on MSC phenotype and that IL-3-treated cells are nonimmunogenic.

### Migration of IL-3-treated MSCs towards SDF-1α

**Fig. 6:**
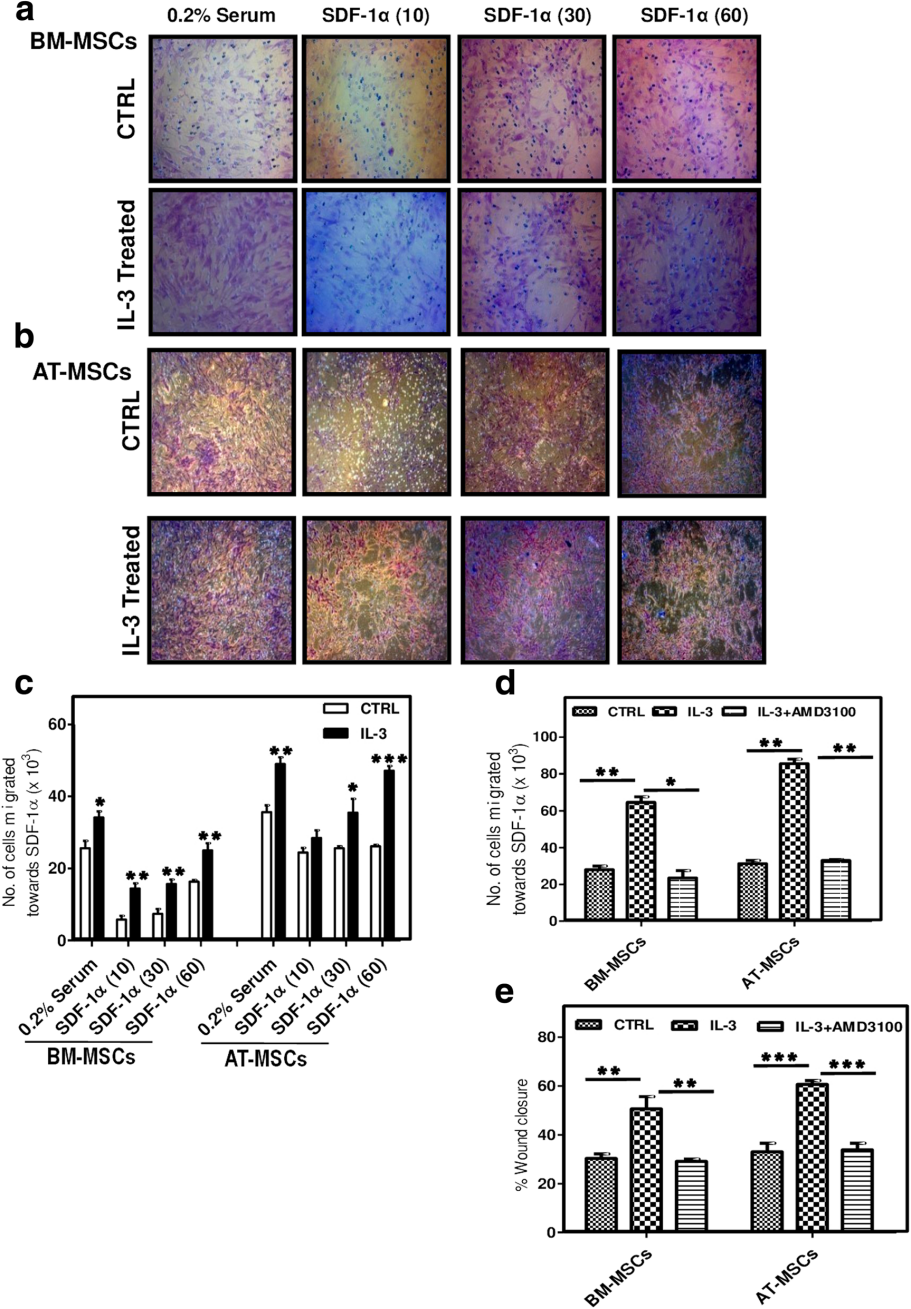
IL-3-treated MSCs migrate towards SDF-1α. IL-3-pretreated (100 ng/ml) and untreated BM-MSCs and AT-MSCs were seeded in the upper chamber of cell culture inserts and SDF-1α (10, 30, 60 ng/ml) was added to the lower chamber. After 18 hours, migration of cells towards SDF-1α was visualized by staining the cells from the lower side of inserts with hematoxylin (**a**, **b**). Cells from the lower side were also removed by trypsinization and counted (**c**). IL-3-pretreated and untreated BM-MSCs and AT-MSCs were seeded in the upper chamber of cell culture inserts with or without AMD3100 (10 μM) and SDF-1α (60 ng/ml) was added to the lower chamber. After 18 hours, cells from the lower side of inserts were trypsinized and counted (**d**). Wounds created on monolayers of BM-MSCs and AT-MSCs were incubated with IL-3 in the presence or absence of AMD3100 and percent wound closure was analyzed (**e**). Data are representative of two independent experiments. *p ≤ 0.05, **p ≤ 0.01 and***p ≤ 0.001 vs control group or vs IL-3-pretreated group. *AT* adipose tissue, *BM* bone marrow, *CTRL* control, *IL-3* interleukin-3, *MSC* mesenchymal stem cell, *SDF-1*α stromal cell-derived factor-1 alpha

CXCR4 is a receptor for SDF-1α and the CXCR4/SDF-1α axis is important for MSC migration and homing [39]. Because IL-3 increases the migration of MSCs through upregulation of CXCR4, we performed a transwell chamber assay to assess cell migration towards SDF-1α. IL-3-treated BM-MSCs and AT-MSCs were added to the upper chamber of cell culture inserts and different concentrations of SDF-1α (10, 30, 60 ng/ml) were added to the lower chamber. We observed that the basal migratory capacity of both MSCs was significantly increased in IL-3-treated cells. Importantly, there was a gradual increase in the migration of IL-3-treated MSCs towards SDF-1α (Fig. 6a, b). Quantitative analysis revealed that a greater number of IL-3-treated MSCs migrated towards SDF-1α in a dose-dependent manner (Fig. 6c), indicating that MSCs with higher CXCR4 expression possess more migratory capacity. Further, addition of CXCR4 antagonist AMD3100 (10 μM) in IL-3-treated MSCs resulted in migration of fewer cells towards SDF-1α (60 ng/ml). This suggests that IL-3-induced migration of MSCs is CXCR4 dependent (Fig. 6d). To further confirm that the effect of IL-3 on migration of MSCs is through CXCR4, we performed a wound healing assay in the presence of AMD3100. We observed that AMD3100 inhibited the migration of IL-3-treated MSCs towards the wound area in both BM-MSCs and AT-MSCs, as evident by the significant decrease in percent wound closure (Fig. 6e). These results suggest that CXCR4/SDF-1α interaction plays a pivotal role in IL-3-induced migration of MSCs.

### In-vivo migration of MSCs towards SDF-1α was enhanced by IL-3

**Fig. 7:**
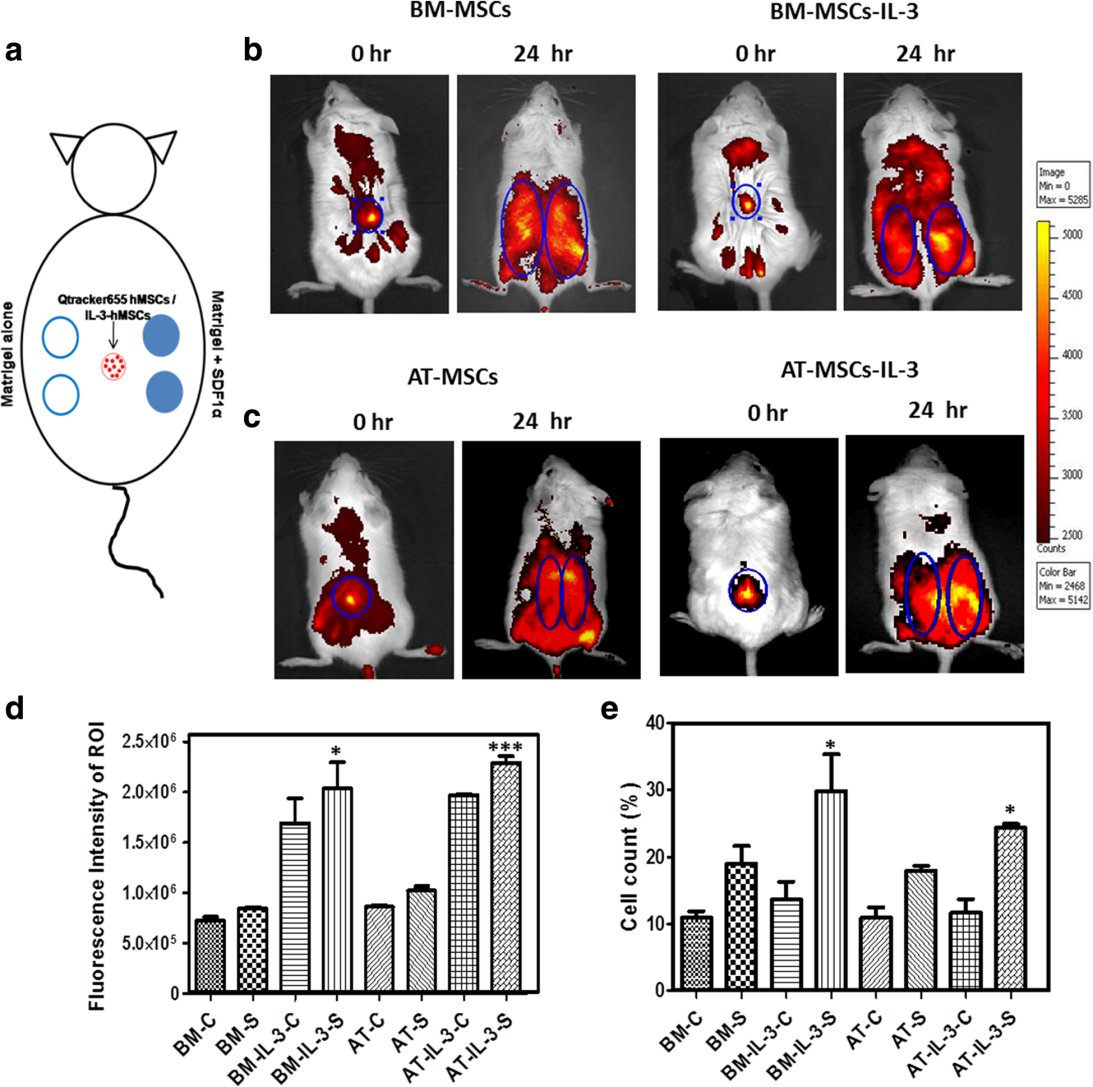
In-vivo migration of IL-3-treated cells towards SDF-1α. Two matrigel plugs mixed with SDF-1α (100 ng/ml) were injected subcutaneously at the right dorsal side of NOD/SCID mice and two matrigel plugs without SDF-1α were injected on the left dorsal side of mice. BM-MSCs and AT-MSCs untreated or pretreated with IL-3 were labeled with Qtracker 655 and injected subcutaneously (10^5^ cells in 100 μl) at the center, equidistant from all four implants. Schematic representation of the subcutaneously implanted matrigel in mouse model (**a**). Mice were acquired on the Live Cell Imaging System at 0 hours for detection of labeled MSCs and at 24 hours for detection of migrated MSCs towards the matrigel plugs (**b**, **c**). Graphical representation showing the counts of fluorescent intensity at the region of interest (*ROI*), the area of matrigel plugs (**d**). Difference between in-vivo migration of IL-3-treated and untreated MSCs towards SDF-1α (BM-IL-3-S vs BM-S and AT-IL-3-S vs AT-S) was compared. Matrigel plugs were harvested from mice and isolated cells were acquired on flow cytometry (**e**). *C* matrigel implant without SDF-1α, *S* matrigel implant containing SDF-1α. Data shown as mean ± SEM (mice, *n* = 6 and matrigel plugs, *n* = 12/group). *p ≤ 0.05 and ***p ≤ 0.001 vs control groups. *AT* adipose tissue, *BM* bone marrow, *IL-3* interleukin-3, *MSC* mesenchymal stem cell, *SDF-1*α stromal cell-derived factor-1 alpha

The in-vitro increase in migration of MSCs towards SDF-1α by IL-3 was further validated in vivo using the SDF-1α-releasing matrigel plug assay in immunocompromised mice. We used matrigel to establish a controlled release of SDF-1α, which is necessary to form a gradient induced migration of MSCs [32]. Matrigel plugs mixed with or without SDF-1α were subcutaneously implanted on the right and left dorsal side of NOD/SCID mice respectively. Qtracker 655-labeled and IL-3-pretreated BM-MSCs and AT-MSCs were injected subcutaneously as described in Methods and as indicated in the schematic representation (Fig. 7a). Mice were acquired on IVIS at 0 hours for detection of labeled MSCs, and at 24 hours for detection of migrated MSCs towards the matrigel plugs. An increased number of IL-3-treated MSCs was detected at SDF-1α-containing matrigel plugs in both the BM-MSC and AT-MSC groups as indicated by fluorescence intensity at the region of interest (ROI) (Fig. 7b, c). Figure 7d represents the average fluorescence intensity of the ROI from three independent experiments which depicts the significant increase in the number of IL-3-treated MSCs in SDF-1α-containing matrigel plugs. Also, flow cytometry analysis of cells isolated from matrigel plugs revealed that IL-3-treated MSCs migrated in greater numbers towards SDF-1α (Fig. 7e). These results suggest that pretreatment of both BM-MSCs and AT-MSCs with IL-3 significantly enhances their migration towards SDF-1α in vivo. Thus, our results suggest that IL-3 increases migration of human MSCs in both in-vitro and in-vivo conditions through the CXCR4/SDF-1α axis.

## Discussion

MSC therapy is considered a promising tool in regenerative medicine for repair of damaged tissues. It is believed that MSCs contribute significantly to wound healing and tissue regeneration by secretion of multiple tropic factors and ultimately differentiate into functional tissue-specific cells [40, 41]. Previously, we and others have reported the beneficial effects of MSC therapy in animal models of various human diseases such as rheumatoid arthritis [2, 40, 42], acute lung injury [43], kidney damage [44] and acute myocardial infarction [45]. MSCs are also known for their immunomodulatory properties primarily mediated by cytokines or regulatory T cells [42, 46]. These multifunctional properties of MSCs indicate their potential in cell-based therapies and regenerative medicine.

Although increasing evidence suggests the beneficial effects and therapeutic use of MSCs for tissue regeneration and recovery, the major obstacle is failure in migration of large numbers of cells towards injured tissues [5]. Various methods are reported for manipulation of MSCs to enhance or improve their recruitment and homing to damaged tissues. One popular approach is to modulate the natural adhesive machinery of MSCs that comprises chemokine receptors and adhesion molecules [24]. Genetic manipulation of preexisting adhesion molecules, membrane modifications, treatment with cytokines and certain chemical factors enhance the expression of molecules required for cell migration [23, 25, 45, 47–49]. Although genetic manipulation of MSCs appears a promising tool to enhance their migration, it may have long-term, unforeseen effects on cellular function and may involve ethical issues and practical difficulties. Pretreatment with cytokines has led to some success in this area; for example, treatment with IFN-γ leads to increased CXCR4 expression and migration of MSCs towards the site of damage in mouse model of colitis [50]. In a similar model, pretreatment with IL-1β is shown to increase migration of MSCs towards the inflammation through upregulation of CXCR4 [25]. However, pretreatment of MSCs with these proinflammatory cytokines may lead to inflammation and cell death in nontargeted tissues and may raise several safety concerns [26]. Also, it is reported that IFN-γ induces the expression of MHC-class II on MSCs, making them more immunogenic [38]. Exposure of MSCs to hypoxia also leads to enhanced migration of cells through modulation of CXCR4; however, these cells may adopt a cancer-like phenotype following hypoxic preconditioning due to accumulation of reactive oxygen species [51]. Amongst all strategies, surface modulation of CXCR4 is widely accepted to help in tissue-directed migration of MSCs mediated by SDF-1α [39, 52–54]. SDF-1α is significantly upregulated in almost all injured tissues, which facilitates the migration and engraftment of circulating CXCR4-positive cells [55]. Therefore, it is believed that inadequate amounts of CXCR4 on the MSC surface may be responsible for the cells’ insufficient migration and homing towards the injured site.

We reported previously that IL-3 inhibits differentiation of human hematopoietic stem cells into bone-resorbing osteoclasts, and also enhances the differentiation of human BM-MSCs into bone-forming osteoblasts under both in-vitro and in-vivo conditions [30, 56]. These results indicated that IL-3 enhances the regenerative potential of human MSCs and is a promising therapeutic candidate for repair or prevention of tissue damage. In the present study, we investigated the role of IL-3 on migration of human MSCs under both in-vitro and in-vivo conditions. Although MSCs are found in many tissues, studies comparing the migration potential of MSCs isolated from different sources are limited. Therefore, we first examined the expression of IL-3Rα on BM-MSCs, AT-MSCs and GT-MSCs and found that all three sources of MSCs express IL-3Rα at gene and protein levels.

Wound healing assay revealed that exposure of MSCs to IL-3 significantly enhances cell migration and wound closure. Cell motility is an important parameter to study the cell migration. Time-lapse microscopic studies of human MSCs in the presence of IL-3 showed significant increase in their motility as evidenced by increased euclidean and accumulated distances. Transendothelial cell migration is a multistep process initiated by firm adherence of cells to the endothelium via selectins, followed by multiple cascades of chemokine and integrin signaling. Human MSCs are known to express a set of chemokine receptors and integrins that are functionally required for cell migration [57]. Engagement of CD44 with its ligand hyaluronic acid or E-selectin induces the firm adhesion and subsequent transendothelial migration [23]. Analysis of chemokine profile of human MSCs showed that IL-3 does not affect the expression of chemokine receptors such as CCR1, CCR7, CCR9, CX3CR1, CXCR5, CXCR6, integrins α4 and α5 and CD44 on MSCs. Interestingly, the expression of CXCR4 was significantly and consistently increased by IL-3 in all three MSCs at both surface as well as intracellular levels. Increased intracellular expression of CXCR4 might serve as a receptor reservoir [58], which can be displayed to cell surface in response to IL-3. Further, real-time PCR analysis revealed 6-fold to 8-fold upregulation of CXCR4 in human MSCs upon IL-3 treatment.

To further confirm the effect of IL-3 on CXCR4 expression, we examined the migration of IL-3-treated MSCs towards SDF-1α. A greater number of IL-3-treated MSCs migrated towards SDF-1α, suggesting its pivotal role in enhancing CXCR4 expression. SDF-1α-mediated migration is inhibited by CXCR4 antagonist AMD3100, indicating the key role of the CXCR4/SDF-1α axis in MSC migration [59]. IL-3-induced CXCR4-mediated migration of MSCs towards SDF-1α was decreased in the presence of AMD3100. Similarly, AMD3100 decreases the migration of IL-3-treated MSCs towards the wound area. These results suggest involvement of the CXCR4/SDF-1α axis in IL-3-induced MSC migration. The therapeutic impact and feasibility of the IL-3 preconditioning approach was evaluated in vivo using a matrigel-releasing SDF-1α implantation assay in NOD/SCID mice. The combination of SDF-1α with matrigel renders the current approach to be of therapeutic relevance, because it generates a SDF-1α gradient, mimicking tissue injury in vivo. Interestingly, IL-3 pretreatment enhances in-vivo migration of MSCs towards SDF-1α.

Comparative study for induction of CXCR4 expression by different cytokines revealed that IL-3 is equally as potent a cytokine as TNF-α and is more potent than IL-1β. Unlike IFN-γ, IL-3 does not alter the phenotypic and immunophenotypic characteristics of MSCs. Additionally, in contrast to TNF-α and IL-1β, IL-3 possesses anti-inflammatory and immunomodulatory properties even in the presence of severe inflammatory conditions and prevents tissue damage in animal models [28, 29, 60]. All of these results suggest that compared to other cytokines IL-3 has the added advantage of its anti-inflammatory and immunomodulatory properties, and it increases both the migration and regenerative potential of human MSCs under both in-vitro and in-vivo conditions. Further we observed that IL-3 does not affect the proliferation of human MSCs and it is not toxic to cells even at higher concentrations (Additional file 1: Figure S2). To further confirm that IL-3 is not toxic in vivo, we evaluated the toxicity of IL-3-treated human MSCs in SCID mice. We found that all hematological parameters and the differential blood cell count were unchanged by infusion of IL-3-treated MSCs (Additional file 1: Table S1). Also, IL-3-treated MSCs did not show any adverse effect on various vital organs (data not shown). In addition, we found that IL-3-treated human MSCs were nontumorogenic in mice (Additional file 1: Figure S3). Thus, IL-3 preconditioning seems to be a promising strategy for improvement of site-directed migration of MSCs and their tissue regeneration potential in vivo. Overall, IL-3 has more therapeutic potential than other cytokines in regenerative cell therapies. Thus, we demonstrate for the first time that pretreatment of MSCs with IL-3 is a novel strategy to achieve better therapeutic outcomes for the cells in tissue regeneration.

## Conclusions

The present study revealed that IL-3 has potential to enhance the migration and motility of human MSCs isolated from different sources. The increased migration of MSCs by IL-3 was mediated through the CXCR4/SDF-1α axis. Interestingly, the IL-3 preconditioning approach enhanced in-vivo migration of MSCs in immunocompromised mice. These results indicate the novel therapeutic role of IL-3 in attaining the efficacy of human MSCs for cell therapy in regenerative medicine.

## Additional file

Additional file 1: Includes supplementary methods, **Figure S1** showing effect of IL-3 on expression of human MSC surface markers, **Figure S2** showing effect of IL-3 on proliferation of human MSCs, **Table S1** presenting hematological parameters of mice infused with IL-3-treated MSCs and **Figure S3** showing tumorigenicity assay of human MSCs pretreated with IL-3. (DOCX 1519 kb)

## Abbreviations

AT: Adipose tissue
BM: Bone marrow
CXCR4: Chemokine (C-X-C motif) receptor 4
GT: Gingival tissue
IL-3: Interleukin-3
MSC: Mesenchymal stem cell
SDF-1α: stromal cell-derived factor-1 alpha

## References

[1] Uccelli A, Moretta L, Pistoia V (2008). Mesenchymal stem cells in health and disease. Nat Rev Immunol.

[2] Djouad F, Bouffi C, Ghannam S, Noel D, Jorgensen C (2009). Mesenchymal stem cells: innovative therapeutic tools for rheumatic diseases. Nat Rev Rheumatol.

[3] Chamberlain G, Fox J, Ashton B, Middlenton J (2007). Concise review: mesenchymal stem cells: their phenotype, differentiation capacity, immunological features, and potential for homing. Stem Cells.

[4] Ma S, Xie N, Li W, Yuan B, Shi Y, Wang Y (2014). Immunobiology of mesenchymal stem cells. Cell Death Differ.

[5] Barbash IM, Chouraqui P, Baron J, Feinberg MS, Etzion S, Tessone A (2003). Systemic delivery of bone marrow-derived mesenchymal stem cells to the infarcted myocardium: feasibility, cell migration, and body distribution. Circulation.

[6] Sarkar D, Spencer JA, Phillips JA, Zhao W, Schafer S, Spelke DP (2011). Engineered cell homing. Blood.

[7] Wang S, Guo L, Ge J, Yu L, Cai T, Tian R (2015). Excess integrins cause lung entrapment of mesenchymal stem cells. Stem Cells.

[8] Pasha Z, Wang Y, Sheikh R, Zhang D, Zhao T, Ashraf M (2008). Preconditioning enhances cell survival and differentiation of stem cells during transplantation in infarcted myocardium. Cardiovasc Res.

[9] Yu SP, Wei Z, Wei L (2013). Preconditioning strategy in stem cell transplantation therapy. Transl Stroke Res.

[10] Devine SM, Cobbs C, Jennings M, Bartholomew A, Hoffman R. Mesenchymal stem cells distribute to a wide range of tissues following systemic infusion into nonhuman primates. Blood. 2003;101:2999–3001. 10.1182/blood-2002-06-1830 12480709

[11] Perez López S, Otero Hernandez, J. Advances in stem cell therapy. In: López-Larrea C, López-Vázquez A, Suárez-Álvarez B, editors. Advances in Experimental Medicine and Biology, New York: Springer; 2012. p. 290–313. 10.1007/978-1-4614-2098-9_19 22457117

[12] François S, Usunier B, Douay L, Benderitter M, Chapel A (2014). Long-term quantitative biodistribution and side effects of human mesenchymal stem cells (hMSCs) engraftment in NOD/SCID mice following irradiation. Stem Cells Int.

[13] Wynn RF, Hart CA, Corradi-Perini C, O’Neill L, Evans C, Wraith J (2004). A small proportion of mesenchymal stem cells strongly expresses functionally active CXCR4 receptor capable of promoting migration to bone marrow. Blood.

[14] Sordi V, Malosio ML, Marchesi F, Mercalli A, Melzi R, Giordano T (2005). Bone marrow mesenchymal stem cells express a restricted set of functionally active chemokine receptors capable of promoting migration to pancreatic islets. Blood.

[15] Granero-moltó F, Weis JA, Miga MI, Landis B, Myers TJ, O’Rear L (2009). Regenerative effects of transplanted mesenchymal stem cells in fracture healing. Stem Cells.

[16] Ji JF, He BP, Dheen ST, Tay SS (2004). Interactions of chemokines and chemokine receptors mediate the migration of mesenchymal stem cells to the impaired site in the brain after hypoglossal nerve injury. Stem Cells.

[17] Lui N, Tian J, Cheng J, Zhang J (2013). Migration of CXCR4 gene-modified bone marrow-derived mesenchymal stem cells to the acute injured kidney. J Cell Biochem.

[18] Wei JN, Cai F, Wang F, Wu XT, Liu L, Hong X (2016). Transplantation of CXCR4 ovrexpressed mesenchymal stem cells augments regeneration in degenerated intervertebral discs. DNA Cell Biol.

[19] Yang J-X, Zhang N, Wang H, Gao P, Yang QP, Wen QP. CXCR4 Receptor overexpression in mesenchymal stem cells facilitates treatment of acute lung injury in rats. J Biol Chem. 2015;290:1994–2006. 10.1074/jbc.M114.605063 PMC4303655 25492872

[20] Kumar S, Ponnazhagan S (2007). Bone homing of mesenchymal stem cells by ectopic alpha 4 integrin expression. FASEB J.

[21] Bobis-Wozowicz S, Miekus K, Wybieralska E, Jarocha D, Zawisz A, Madeja Z (2011). Genetically modified adipose tissue-derived mesenchymal stem cells overexpressing CXCR4 display increased motility, invasiveness, and homing to bone marrow of NOD/SCID mice. Exp Hematol.

[22] Guan M, Yao W, Liu R, Lam KS, Nolta J, Jia J (2012). Directing mesenchymal stem cells to bone to augment bone formation and increase bone mass. Nat Med.

[23] Thankamony SP, Sackstein R (2011). Enforced hematopoietic cell E-and L-selectin ligand (HCELL) expression primes transendothelial migration of human mesenchymal stem cells. Proc Natl Acad Sci U S A.

[24] Ponte AL, Marais E, Gallay N, Langonne A, Delorme B, Herault O (2007). The in vitro migration capacity of human bone marrow mesenchymal stem cells: comparison of chemokine and growth factor chemotactic activities. Stem Cells.

[25] Fan H, Zhao G, Liu L, Liu F, Gong W, Liu X (2012). Pre-treatment with IL-1β enhances the efficacy of MSC transplantation in DSS-induced colitis. Cell Mol Immunol.

[26] Liu Y, Wang L, Kikuiri T, Akiyama K, Chen C, Xu X, et al. Mesenchymal stem cell-based tissue regeneration is governed by recipient T lymphocytes via IFN-γ and TNF-α. Nat Med. 2011;17:1594–601. 10.1038/nm.2542 PMC3233650 22101767

[27] Shi M, Li J, Liao L, Chen B, Li B, Chen L, et al. Regulation of CXCR4 expression in human mesenchymal stem cells by cytokine treatment: role in homing efficiency in NOD/SCID mice. Haematologica. 2007;92:897–904. 10.3324/haematol.10669 17606439

[28] Srivastava RK, Tomar GB, Barhanpurkar AP, Gupta N, Pote ST, Mishra GC (2011). IL-3 attenuates collagen-induced arthritis by modulating the development of Foxp3+ regulatory T cells. J Immunol.

[29] Kour S, Garimella MG, Shiroor DA, Mhaske ST, Joshi SJ, Singh K (2016). IL-3 decreases cartilage degeneration by downregulating matrix metalloproteinases and reduces joint destruction in osteoarthritic mice. J Immunol.

[30] Barhanpurkar AP, Gupta N, Srivastava RK, Tomar GB, Naik SP, Joshi SR (2012). IL-3 promotes osteoblast differentiation and bone formation in human mesenchymal stem cells. Biochem Biophys Res Commun.

[31] Tomar GB, Srivastava RK, Gupta N, Barhanpurkar A, Pote ST, Jhaveri HM (2010). Human gingiva-derived mesenchymal stem cells are superior to bone marrow-derived mesenchymal stem cells for cell therapy in regenerative medicine. Biochem Biophys Res Commun.

[32] Naderi-Meshkin H, Martin MM, Heirani-Tabasi A, Mirahmadi M, Irfan-Maqsood M, Edalatmanesh MA (2016). Injectable hydrogel delivery plus preconditioning of mesenchymal stem cells: exploitation of SDF-1/CXCR4 axis towards enhancing the efficacy of stem cells’ homing. Cell Biol Int.

[33] Lv FJ, Tuan RS, Cheung KM, Leung VY (2014). Concise review: the surface markers and identity of human mesenchymal stem cells. Stem Cells.

[34] Huang H, Kim HJ, Chang EJ, Lee ZH, Hwang SJ, Kim H-M (2009). IL-17 stimulates the proliferation and differentiation of human mesenchymal stem cells: implications for bone remodelling. Cell Death Differ.

[35] Liu X, Wang Z, Wang R, Zhao F, Shi P, Jiang Y, Pang X (2013). Direct comparison of the potency of human mesenchymal stem cells derived from amnion tissue, bone marrow and adipose tissue at inducing dermal fibroblast responses to cutaneous wounds. Int J Mol Med.

[36] Haasters F, Prall WC, Westphal I, Bocker W, Padula D, Mutschler W (2013). Overexpression of dnIKK in mesenchymal stem cells leads to increased migration and decreased invasion upon TNF-stimulation. Biochem Biophys Res Commun.

[37] Sivanathan KN, Rojas-Canales DM, Hope CM, Krishnan R, Carroll RP, Gronthos S (2015). Interleukin-17A-induced human mesenchymal stem cells are superior modulators of immunological function. Stem Cells.

[38] Sivanathan KN, Gronthos S, Rojas-Canales D, Thierry B, Coates PT (2014). Interferon-gamma modification of mesenchymal stem cells: implications of autologous and allogeneic mesenchymal stem cell therapy in allotransplantation. Stem Cell Rev Reports.

[39] Kitaori T, Ito H, Schwarz EM, Tsutsumi R, Yoshitomi H, Oishi S (2009). Stromal cell-derived factor 1/CXCR4 signaling is critical for the recruitment of mesenchymal stem cells to the fracture site during skeletal repair in a mouse model. Arthritis Rheum..

[40] Maumus M, Jorgensen C, Noël D (2013). Mesenchymal stem cells in regenerative medicine applied to rheumatic diseases: role of secretome and exosomes. Biochimie.

[41] Wang Z, Wang Y, Wang Z, Gutkind JS, Wang Z, Wang F (2015). Engineered mesenchymal stem cells with enhanced tropism and paracrine secretion of cytokines and growth factors to treat traumatic brain injury. Stem Cells.

[42] Garimella MG, Kour S, Piprode V, Mittal M, Kumar A, Rani L (2015). Adipose-derived mesenchymal stem cells prevent systemic bone loss in collagen-induced arthritis. J Immunol.

[43] Matthay M, Goolaerts A, Howard JP, Lee JW (2010). Mesenchymal stem cells for acute lung injury: preclinical evidence. Crit Care.

[44] Barnes CJ, Distaso CT, Spitz KM, Verdun VA, Haramati A (2016). Comparison of stem cell therapies for acute kidney injury. Am J Stem Cells.

[45] Kraitchman DL, Tatsumi M, Gilson WD, Ishimori T, Kedziorek D, Walczak P (2005). Dynamic imaging of allogeneic mesenchymal stem cells trafficking to myocardial infarction. Circulation.

[46] Wang Y, Chen X, Cao W, Shi Y (2014). Plasticity of mesenchymal stem cells in immunomodulation: pathological and therapeutic implications. Nat Immunol.

[47] Haider HK, Jiang S, Idris NM, Ashraf M (2008). IGF-1-overexpressing mesenchymal stem cells accelerate bone marrow stem cell mobilization via paracrine activation of SDF-1α/CXCR4 signaling to promote myocardial repair. Circ Res.

[48] Cho SW, Sun HJ, Yang JY, Jung JY, An JH, Cho HY (2009). Transplantation of mesenchymal stem cells overexpressing RANK-Fc or CXCR4 prevents bone loss in ovariectomized mice. Mol Ther.

[49] Deveza L, Choi J, Lee J, Hunag N, Cooke J, Yang F (2016). Polymer-DNA nanoparticle-induced CXCR4 overexpression improves stem cell engraftment and tissue regeneration in a mouse hindlimb ischemia model. Theranostics.

[50] Duijvestein M, Wildenberg ME, Welling MM, Hennink S, Molendijk I, Zuylen VL (2011). Pretreatment with interferon-g enhances the therapeutic activity of mesenchymal stromal cells in animal models of colitis. Stem Cells.

[51] Crowder SW, Horton LW, Lee SH, McClain CM, Hawkins OE, Palmer AM (2013). Passage-dependent cancerous transformation of human mesenchymal stem cells under carcinogenic hypoxia. FASEB J.

[52] Cheng Z, Ou L, Zhou X, Li F, Jia X, Zhang Y (2008). Targeted migration of mesenchymal stem cells modified with CXCR4 gene to infarcted myocardium improves cardiac performance. Mol Ther.

[53] Karp JM, Leng Teo GS (2009). Mesenchymal stem cell homing: the Devil is in the details. Cell Stem Cell.

[54] Jones GN, Moschidou D, Lay K, Abdulrazzak H, Vanleene M, Shefelbine S (2012). Upregulating CXCR4 in human fetal mesenchymal stem cells enhances engraftment and bone mechanics in a mouse model of osteogenesis imperfecta. Stem Cells Transl Med.

[55] Li L, Jiang J (2011). Regulatory factors of mesenchymal stem cell migration into injured tissues and their signal transduction mechanisms. Front Med.

[56] Gupta N, Barhanpurkar AP, Tomar GB, Srivastava RK, Kaur S, Pote ST (2010). IL-3 Inhibits human osteoclastogenesis and bone resorption through downregulation of c-Fms and diverts the cells to dendritic cell lineage. J Immunol.

[57] Honczarenko M, Le Y, Swierkowski M, Ghiran I, Glodek AM, Silberstein LE (2006). Human bone marrow stromal cells express a distinct set of biologically functional chemokine receptors. Stem Cells.

[58] Kollet O, Petit I, Kahn J, Samira S, Dar A, Pele A (2002). Human CD34^+^CXCR4^–^ sorted cells harbor intracellular CXCR4, which can be functionally expressed and provide NOD/SCID repopulation. Blood.

[59] Liu N, Patzak A, Zhang J (2013). CXCR4-overexpressing bone marrow-derived mesenchymal stem cells improve repair of acute kidney injury. Am J Physiol Renal Physiol.

[60] Yogesha SD, Khapli SM, Srivastava RK, Mangashetti LS, Pote ST, Mishra GC (2009). IL-3 inhibits TNF-alpha-induced bone resorption and prevents inflammatory arthritis. J Immunol.

