# Supplementary material for: Interleukin-3 enhances the migration of human mesenchymal stem cells by regulating expression of CXCR4
Source: Stem Cell Res Ther. 2017 Jul 14;8:168. doi: 10.1186/s13287-017-0618-y (PMC5512829; doi:10.1186/s13287-017-0618-y)
Supplement: Additional file 1: — Includes supplementary methods, Figure S1 showing effect of IL-3 on expression of human MSC surface markers, Figure S2 showing effect of IL-3 on proliferation of human MSCs, Table S1 presenting hematological parameters of mice infused with IL-3-treated MSCs and Figure S3 showing tumorigenicity assay of human MSCs pretreated with IL-3. (DOCX 1519 kb) [file 13287_2017_618_MOESM1_ESM.docx]

**Additional file 1**

**Supplementary Methods**

**MTT assay**

Human BM-MSCs, AT-MSCs and GT-MSCs were cultured in 96 well plates (2 x 10^3^ cells/well) and incubated with IL-3 at concentrations of 1, 10 and 100 ng/ml for 6 days. After 6 days, cell culture media was replaced with 100 μl of MTT solution and was further incubated at 37^o^C for 3-4 hours. After 4 hours the MTT solution was discarded and replaced with 100 μl of MTT solvent and incubated at room temperature for 5 minutes. The formazon crystals were dissolved by thoroughly mixing the solution with pipette and absorbance was measured at 570 nm.

**In vivo toxicity study of human MSCs treated with IL-3**

Human MSCs (2.5 x 10^5^ cells/mouse) were treated with human recombinat IL-3 (100 ng/ml) for 24 hours and then injected intravenously into SCID mice. Mice were observed for 6 days for clinical signs of illness, skin lesions and mortality if any. On day 7, mice were sacrificed for analysis of hematological parameters and histopathological studies. Hematological parameters were analyzed using Sysmex XP-100 (Transasia) Autoanalyzer. Differential blood count on blood films was performed by Leishman's staining**.**

**In vivo tumorogenicity assay**

Human BM-MSCs and AT-MSCs (2 x 10^6^ cells/mouse) treated with IL-3 were mixed with the Matrigel and implanted subcutaneously into SCID mice for 6 months. HeLa (human epithelial carcinoma) cells were used as a positive control for tumor formation.

**Figure S1**


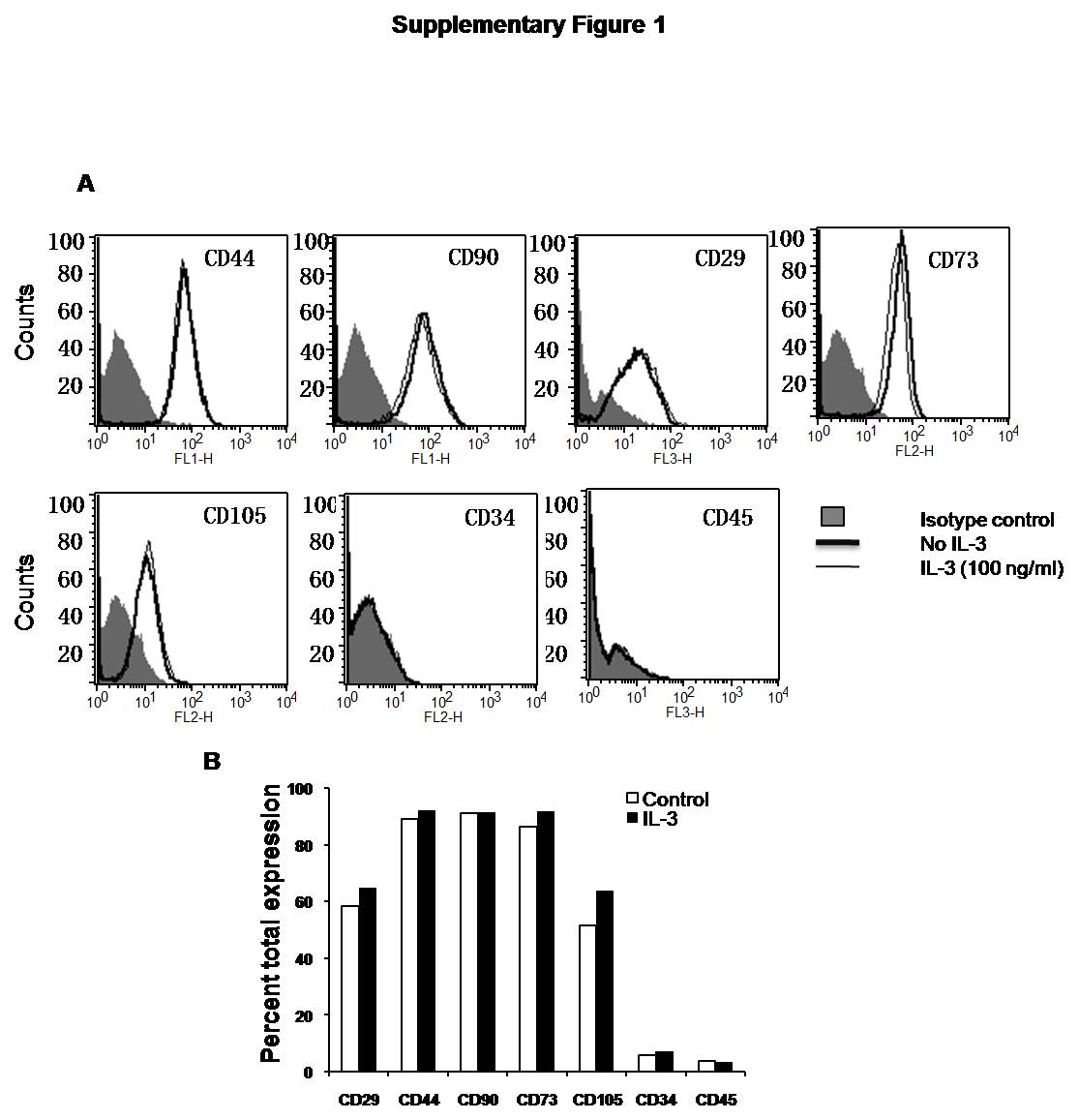


Figure S1. Effect of IL-3 on expression of human MSC surface markers. Human BM-MSCs plated at a density of 1.25 x 10^5^ cells/T-25 cm^2^ flask were incubated with and without IL-3 (100 ng/ml) for 7 days. MSC surface markers expression was analyzed by flow cytometry by staining the cells with CD29, CD44, CD90, CD73, CD105, CD34 and CD45 antibodies and their respective isotypes (**A**). The graph was plotted as percent total expression of MSC markers (**B**). Similar results were obtained in two independent experiments.

**Figure S2**


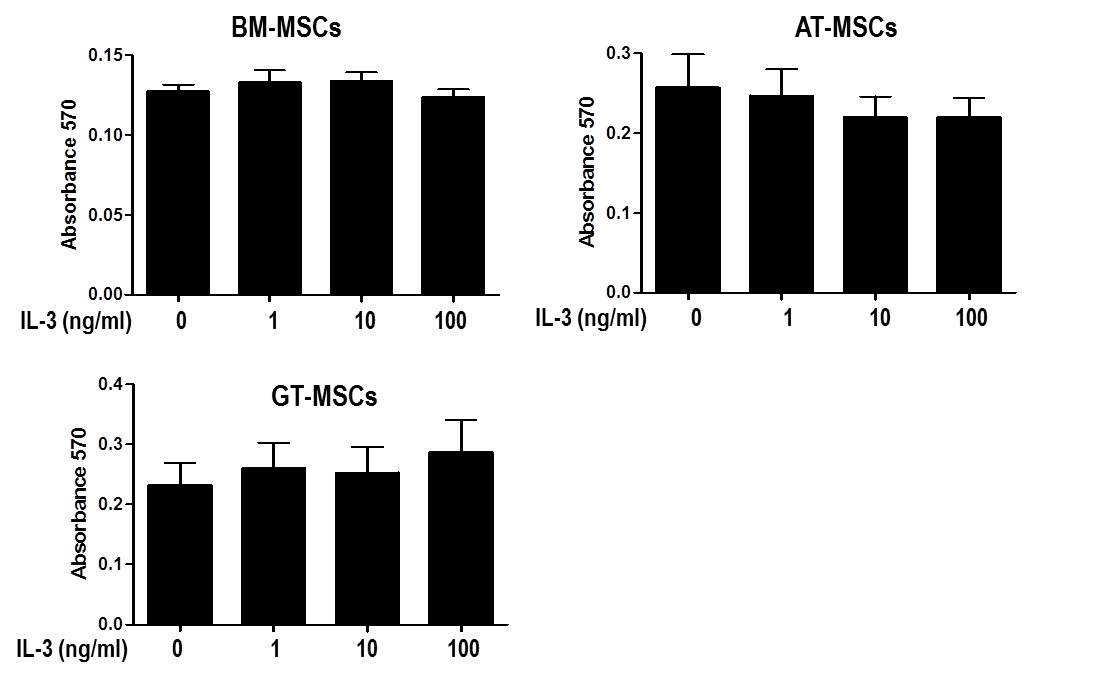


Figure S2. Effect of IL-3 on proliferation of human MSCs. Human BM-MSCs, AT-MSCs and GT-MSCs (2 x 10^3^  cells/well) were seeded in 96 well plates and incubated for 6 days with different concentrations of IL-3 (1, 10 and 100 ng/ml). Cell proliferation was assessed by MTT assay. Data is representative of three independent experiments.

**Table S1**

**Hematological parameters of mice infused with IL-3 treated MSCs.**

|  | **PBS injected control mice** | **Control-MSCs injected mice** | **IL-3-MSCs injected mice** |
| --- | --- | --- | --- |
| **WBC (10^3^/µl)** | **3.433 ± 0.6** | **3.85 ± 0.9** | **3.5 ± 1.1** |
| **RBC (10^6^/µl)** | **9.387 ± 1.1** | **8.055 ± 0.4** | **7.99 ± 0.6** |
| **HGB (g/dL)** | **12.3 ± 0.4** | **12.35 ± 0.1** | **12.52 ± 0.7** |
| **HCT (%)** | **44.57 ± 1.5** | **45.7 ± 0.2** | **45.7 ± 2.4** |
| **MCV (fL)** | **55.33 ± 1.1** | **56.8 ± 1.2** | **57.26 ± 1.6** |
| **MCH (pg)** | **15.23 ± 0.3** | **15.35 ± 0.6** | **15.7 ± 0.6** |
| **MCHC (g/dL)** | **27.6 ± 0.2** | **27.03 ± 0.2** | **27.42 ± 0.5** |
| **PLT (10^3^/µl)** | **1605 ± 0.4** | **1427 ± 2.5** | **1516 ± 2.5** |
| **Eosinophils (%)** | **1.5 ± 0.70** | **1.5 ± 0.57** | **1.2 ± 0.84** |
| **Basophils (%)** | **0.75 ± 0.35** | **0.5 ± 0.58** | **0.6 ± 0.55** |
| **Neutrophils (%)** | **73 ± 5.66** | **69.75 ± 2.99** | **69.2 ± 4.09** |
| **Lymphocytes (%)** | **22.5 ± 4.95** | **23.75 ± 4.03** | **24.6 ± 4.62** |
| **Monocytes (%)** | **4.5 ± 0.71** | **4.5 ± 1.29** | **4.4 ± 1.51** |

Human MSCs pre-treated with recombinant human IL-3 (100 ng/ml) for 24 hours were infused intravenously (2.5 x 10^5^ cells/mouse) into SCID mice (n = 5). After 7 days blood was collected from orbital plexus into EDTA vaccutainers and hematological parameters were analyzed using Sysmex XP-100 (Transasia) Autoanalyzer. Differential blood count on blood films was performed by Leishman's staining**.**

**Figure S3**

**
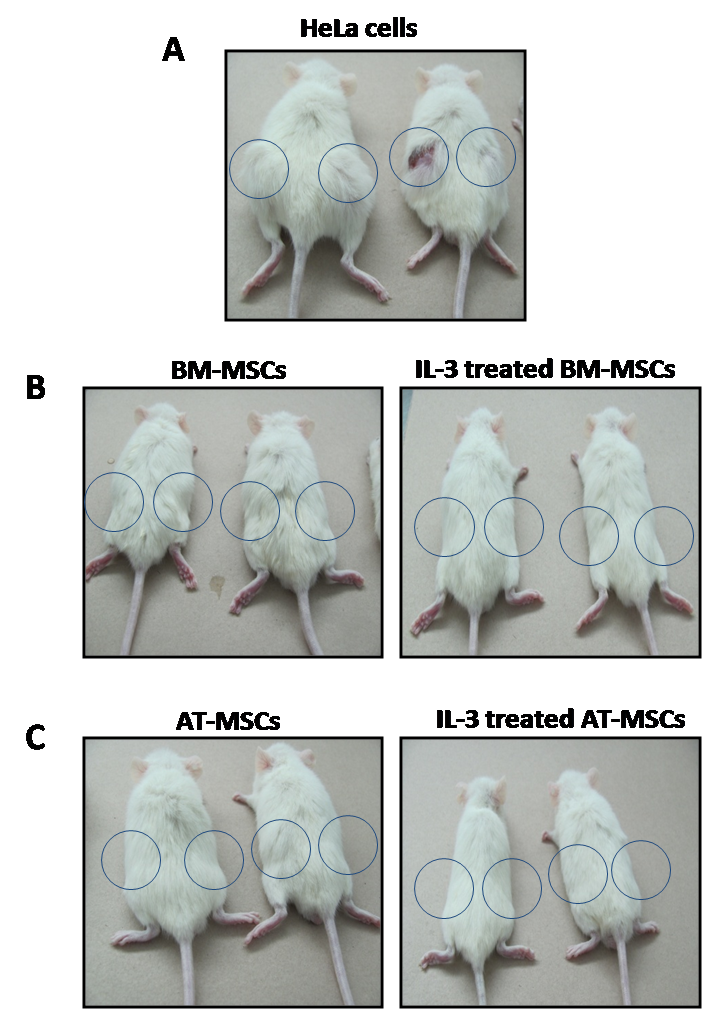
**

Figure S3:Tumorigenecity assay of human MSCs pre-treated with IL-3. Human BM-MSCs and AT-MSCs (2 x 10^6^ cells/ mouse) were pre-treated with IL-3 (100 ng/ml, 24 hrs) and mixed with Matrigel. Cells mixed with matrigel were implanted subcutaneously into NOD/SCID mice and were observed for 6 months. HeLa cells were used as a positive control for tumor formation. Photographs of HeLa cells injected mice after 3 weeks (A). Photographs of BM-MSCs (B) and AT-MSCs (C) injected mice after 6 months. The circles indicate the site of injection of MSCs.
